# C-751-04. Histological Long-Term Evaluation of Scars After STSG versus Dermal Template (DRT) + STSG

**DOI:** 10.1093/jbcr/irag033.009

**Published:** 2026-04-06

**Authors:** Sigrid Blome-Eberwein, Sakura Helm

**Affiliations:** Lehigh Valley Health Network Regional Burn Center, Allentown, PA; Lehigh Valley Health Network Regional Burn Center, Allentown, PA

## Abstract

**Introduction:**

Dermal scaffolds (DRT) have been used to assist in (burn) wound closure for decades, yet long term evaluation of scar outcomes are scarce. A standardized histological scar scoring system in general is not available. Some clinical parameters have been studied and published, but a thorough, controlled, clinical and histological study has not been performed in humans to date.

**Methods:**

We invited long term burn survivors who received both, plain STSG and dermal scaffold plus STSG to close their wounds. Clinical measurements and POSAS evaluation was performed and biopsies were obtained from scars with and without dermal scaffold underlay. The biopsies were processed, stained and analyzed. A subset of patients’ samples (n = 6 for STSG and n = 5 DRT + STSG, n = 4 were matched samples) were analyzed using an extracellular matrix pattern Fiji (ImageJ) plugin, TWOMBLI to include semi-quantitative analysis of collagen fiber alignment, length, branching, endpoints, fractal dimension (measure of fiber complexity and organization), curvature, and distribution of fiber thickness (Wershof et al. 2021 Life Sci Alliance). Additional analysis was performed in Fiji (ImageJ) including measuring average width of epidermis and average length of rete pegs. We developed a new scoring system for histologic scar evaluation, that is being validated.

**Results:**

While semi-quantitative histological analysis illustrated no statistical differences between the STSG alone and DRT + STSG scars for the metrics listed above in this small subset, we were able to note consistent differences in structure scoring. Some scars had evidence of intact rete pegs (4/6 for STSG samples, and 2/4 for DRT + STSG samples) (Fig. 1), which are typically diminished or non-apparent in post-burn scars (Carney et al 2024 Burns). Rete peg length ranged from 50-200um. In Fig. 1, the epidermis appears thicker and dermis appears less aligned for the STSG, relative to the DRT + STSG group where the epidermis appears thinner with a more aligned dermis. Scar scales and non-invasive quantitative measures of scar characteristic were previously reported and will be matched with histological findings.

**Conclusions:**

In this small subset of available analyzed histologies differences can be seen between STSG scars and DRT + STSG scars years after wound healing in the same survivor. The further development and validation of our scar scoring system will highlight these differences further. Matched with the clinical data that were collected from the same individuals, we will validate our findings.

**Applicability of Research to Practice:**

Characterization and objective evaluation of long term outcomes of burn scars that healed with different technologies.

**Funding for the study:**

Foundation funding.

